# Arthrogenicity of type II collagen monoclonal antibodies associated with complement activation and antigen affinity

**DOI:** 10.1186/1476-9255-8-31

**Published:** 2011-11-04

**Authors:** Thongchai Koobkokkruad, Tatsuya Kadotani, Pilaiwanwadee Hutamekalin, Nobuaki Mizutani, Shin Yoshino

**Affiliations:** 1 Department of Pharmacology, Kobe Pharmaceutical University, 4-9-1 Motoyamakita-machi, Higashinada-ku, Kobe-shi, Hyogo-ken, Japan

## Abstract

**Background:**

The collagen antibody-induced arthritis (CAIA) model, which employs a cocktail of monoclonal antibodies (mAbs) to type II collagen (CII), has been widely used for studying the pathogenesis of autoimmune arthritis. In this model, not all mAbs to CII are capable of inducing arthritis because one of the initial events is the formation of collagen-antibody immune complexes on the cartilage surface or in the synovium, and subsequent activation of the complement by the complexes induces arthritis, suggesting that a combination of mAbs showing strong ability to bind mouse CII and activate the complement may effectively induce arthritis in mice. In the present study, we examined the relationship between the induction of arthritis by the combination of IgG2a (CII-6 and C2A-12), IgG2b (CII-3, C2B-14 and C2B-16) and IgM (CM-5) subclones of monoclonal antibodies (mAb) of anti-bovine or chicken CII and the ability of mAbs to activate complement and bind mouse CII.

**Methods:**

DBA/1J mice were injected with several combinations of mAbs followed by lipopolysaccharide. Furthermore, the ability of mAbs to activate the complement and bind mouse CII was examined by ELISA.

**Results:**

First, DBA/1J mice were injected with the combined 4 mAbs (CII-3, CII-6, C2B-14, and CM-5) followed by lipopolysaccharide, resulting in moderate arthritis. Excluding one of the mAbs, i.e., using only CII-3, CII-6, and C2B-14, induced greater inflammation of the joints. Next, adding C2A-12 but not C2B-16 to these 3 mAbs produced more severe arthritis. A combination of five clones, consisting of all 5 mAbs, was less effective. Histologically, mice given the newly developed 4-clone cocktail had marked proliferation of synovial tissues, massive infiltration by inflammatory cells, and severe destruction of cartilage and bone. Furthermore, 4 of the 6 clones (CII-3, CII-6, C2B-14, and C2A-12) showed not only a strong cross-reaction with mouse CII but also marked activation of the complement *in vitro*.

**Conclusion:**

The combination of 4 mAbs showing strong abilities to activate the complement and bind mouse CII effectively induced arthritis in DBA/1J mice. This *in vitro *system may be useful for the selection of mAbs associated with the development of arthritis.

## Background

Rheumatoid arthritis (RA) is an autoimmune disease characterized by chronic inflammation of the joints and the subsequent destruction of cartilage and bone associated with elevated levels of autoantibodies to type II collagen (CII) in both cartilage and synovium [1,2]. The most commonly used animal model for RA is collagen-induced arthritis (CIA), showing chronic inflammation of multiple joints, induced by immunizing rodents with CII [3-5]. In patients with RA [6] and the CIA model [7-9], increased levels of complement C3a in serum have been described [10-14], suggesting that the activation of complement-producing pathways through antigen-antibody immune complexes regulates arthritis.

Arthritis similar to that in the CIA model can be induced in naïve mice by transferring serum containing autoantibodies to CII from arthritic mice [15]. Furthermore, the collagen antibody-induced arthritis (CAIA) model, which employs a cocktail of monoclonal antibodies (mAbs) to CII, has been widely used for studying the pathogenesis of autoimmune arthritis and evaluating therapeutics [16-18]. It is an exceedingly valuable tool because consistent and severe arthritis can be induced within days instead of the 4 weeks required to induce CIA in mice [19]. On the other hand, not all mAbs to CII are capable of inducing arthritis because the initial event in this model is the formation of collagen-antibody immune complexes on the cartilage surface or in the synovium, and subsequent activation of the complement by the complexes may induce arthritis, suggesting that a combination of mAbs showing strong ability to bind mouse CII and activate the complement may effectively induce arthritis in mice; however, the relationship between the development of arthritis and the ability of mAbs to activate complement and bind mouse CII has not fully been examined.

We have previously developed IgG2a (CII-6) and IgG2b (CII-3) subtypes of anti-CII mAbs from spleen cells of DBA/1J mice immunized with bovine CII (Hutamekalin et al., 2009). In the present study, we developed IgG2a (C2A-12), IgG2b (C2B-14 and C2B-16), and IgM (CM-5) subtypes of anti-CII mAbs from DBA/1J mice immunized with chicken CII. Therefore, we examine whether arthritis is induced by i.p. injection with several combinations of anti-CII mAbs followed by lipopolysaccharide (LPS), shown to exacerbate arthritis in both CIA [20] and CAIA models [16,17]. Furthermore, to examine the relationship between the development of arthritis and the ability of mAbs to activate the complement and bind mouse CII, we measured cross-reactions with mouse CII and activation of the complement *in vitro*.

## Materials and methods

### Animals

Male DBA/1J mice (8 weeks of age) were bred in the animal breeding unit of Kobe Pharmaceutical University, Kobe, Japan. The mice were housed in a specific pathogen-free environment and fed standard rodent chow and water *ad libitum*. All procedures were performed with the approval of the Institutional Animal Care and Use Committee.

### mAbs to CII

In this study, we developed IgG2a (C2A-12), IgG2b (C2B-14 and C2B-16) and IgM (CM-5) subtypes of anti-CII mAbs from spleen cells of DBA/1J mice immunized with chicken CII (Sigma-Aldrich Fine Chemicals, MI, USA) emulsified with CFA (Difco Laboratories, Detroit, MI, USA) as described previously [16,18]. Briefly, mice were given a booster injection of 0.1 mg chicken CII dissolved in 100 μl JG buffer on days 11-13. Three days after the injection, spleen cells (1 × 10^8^) were obtained and fused with NS-1 myeloma cells (2 × 10^7^) using PEG1500 (Roche Diagnostics GmbH, Mannheim, Germany) according to the manufacturer's instructions.

Hybridoma cells producing antibodies against chicken CII were screened by ELISA using plates coated with chicken CII (10 μg/ml in JG buffer). The wells were blocked with 1% casein (Sigma-Aldrich) dissolved in PBS at room temperature for 1 h. Fifty microliters of culture medium mixed with an equal volume of PBS containing 1% Tween 20 (Sigma-Aldrich) was reacted at 37°C for 1 h. mAbs bound to collagen were detected by phosphatase-labeled anti-mouse IgG (Fc) (Sigma-Aldrich). Color was developed by adding 100 μl of 3 mM *p-*nitrophenylphosphate (Bio-Rad, Richmond, CA, USA), and absorbance was measured at 405 nm using an IMMUNO-MINI NJ-2300 (Thermo Fisher Scientific, Roskilde, Denmark).

The selected hybridoma cells were cloned by limited dilution and cultured in a serum-free CM-B medium (Sanko Junyoku Co. Ltd., Tokyu, Japan) in nunc™ 96-microwell plates (Thermo Fisher Scientific). mAbs were purified by HiTrap IgG Protein A or HiTrap IgM (GE Healthcare, Uppsala, Sweden) affinity chromatography, and concentrated by Vivaspin-20 (Sartorius Stedim Biotech Gmbh, Goettingen, Germany) to 10 mg/ml in PBS based on an OD280 of IgG mAb at 1 mg/ml of 1.42.

### Induction of arthritis

The 3-or 4-clone cocktail was prepared by mixing an equal volume of 10 mg/mL, and mice were given 0.6 or 0.8 mL of the cocktail (6 or 8 mg/mouse) by i.p. injection on day 0, respectively, followed by an i.p. injection of LPS (50 μg/mouse) on day 3.

The mice were observed daily after the injection of mAbs for the development of arthritis until day 10. The severity of arthritis was scored as: 0 = normal; 1 = mild erythema or swelling of wrist or ankle or erythema and swelling of any severity for 1 digit; 2 = more than three inflamed digits or moderate erythema and swelling of the ankle or wrist; 3 = severe erythema and swelling inflammation of wrist or ankle; 4 = complete erythema and swelling of the wrist and ankle including all digits.

### Histopathology and immunohistochemistry assessment of arthritis

Front paw joints were dissected on day 10, fixed in 10% neutral-buffered formalin, decalcified in decalcifying solution (Wako, Osaka, Japan), and embedded in paraffin. The front ankle joints were sectioned at 4 μm and stained with hematoxylin and eosin (H&E) by the standard technique.

For immunohistochemical staining, the sections were deparaffinized and hydrated through xylene and a graded alcohol series. The sections were depleted of endogenous peroxidase by incubating in 3% H_2_O_2 _in distilled water for 30 min. After blocking non-specific binding with diluted normal rabbit or goat serum in PBS for 20 min, the sections were incubated for 1 h at room temperature with a primary antibody against IL-1beta (SC-1251, goat IgG; Santa Cruz Biotechnology, Santa Cruz, CA) or TNF-alpha (HP8001, rabbit IgG; Hycult Biotechnology BV, Uden, Netherlands). The sections for IL-1beta and TNF-alpha were developed using a VECTASTAIN Elite ABC goat kit and rabbit IgG kit, respectively, and a DAB substrate kit for peroxydase (Vector Laboratories, South San Francisco, CA). Counterstaining was performed with hematoxylin. As a negative control, goat or rabbit IgG was used.

### Activation of C3 *in vitro *by mAbs

The activation of C3 *in vitro *by mAbs (CII-6, C2A-12, CII-3, C2B-14, C2B-16, and CM-5) was examined by ELISA with modification of the system developed by Banda et al. [12]. Dilutions (100-800 μg/ml) of mAbs were detected using plates coated with chicken CII (25 μg/ml) and adding complement (Rockland Immunochemicals, PA). Horseradish peroxidase-conjugated goat IgG anti-mouse C3 antibody (MP Biomedical, OH, USA) was added and the color reaction was examined by adding TMB substrate (BD Pharmingen, MA, USA) at 450 nm using a microplate reader. Values for the activation of C3 by mAbs were expressed as a percentage of the CII-3 value (800 μg/ml).

### Cross-reaction of mAbs with mouse or chicken CII

The cross-reaction of mAbs (CII-6, C2A-12, CII-3, C2B-14, C2B-16, and CM-5) with mouse or chicken CII (1 μg/ml) was determined by ELISA with affinity for collagen. Dilutions (0.001-1000 μg/ml) of mAbs were detected using plates coated with mouse or chicken CII and adding phosphate-labeled anti-mouse IgG (Fc) or IgM (Sigma-Aldrich). The plates were developed with *p-*nitro phenyl phosphatase and read at 405 nm using a microplate reader. Values for the cross-reaction of mAbs with mouse or chicken CII were expressed as a percentage of the CII-3 value (1000 μg/ml).

## Results

### Time course of changes in the arthritis score induced by arthritogenic mAbs

**Figure 1:**
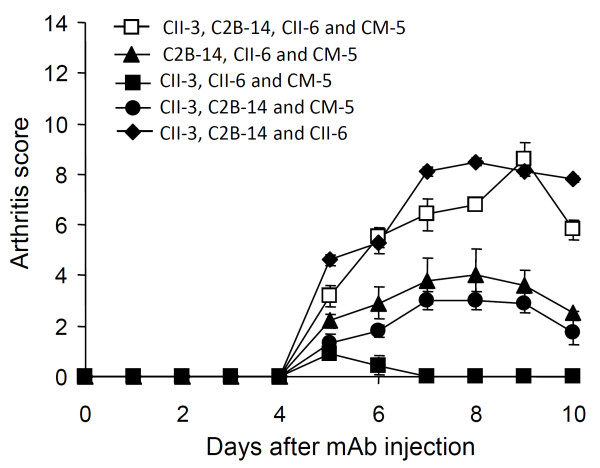
**Time course of changes in the arthritic score after the administration of arthritogenic mAbs**. DBA/1J mice received i.p. injections of 4 clones (CII-3, C2B-14, CII-6 and CM-5), 3 clones (C2B-14, CII-6 and CM-5), 3 clones (CII-3, CII-6 and CM-5), 3 clones (CII-3, C2B-14 and CM-5), and 3 clones (CII-3, C2B-14 and CII-6) on day 0 followed by LPS. Each value is the mean ± SEM for five animals.

First, we investigated whether arthritis is induced by combinations of CII-3, CII-6, C2B-14, and CM-5 in DBA/1J mice (Figure 1). The 4 mAbs combined caused arthritis, the severity of which was 6.8 ± 0.2 on day 8. Furthermore, a cocktail of 3 mAbs (CII-3, CII-6, and C2B-14) induced greater inflammation of the joints than any other combination (arthritic score: 8.5 ± 0.2 on day 8). On the other hand, the combination of CII-3, CII-6, and CM-5 (without C2B-14) caused no arthritis.

Consequently, the combination of CII-3, CII-6, and C2B-14 was used in subsequent experiments.

### Effect of an extra mAb on the arthritogenicity of the 3-clone cocktail

**Figure 2:**
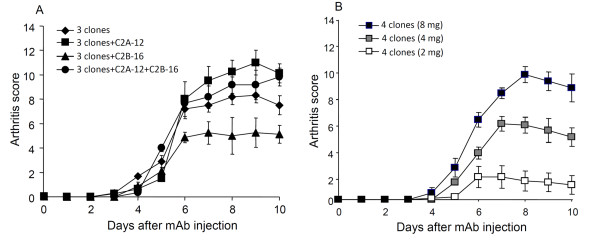
**Effect of an extra monoclonal antibody on the arthritogenicity of the 3-clone cocktail (CII-3, C2B-14, and CII-6)**. **A**: DBA/1J mice were given i.p. injections of a cocktail of CII-3, C2B-14, and CII-6, the cocktail plus C2A-12, the cocktail plus C2B-16 and the cocktail plus C2A-12 and C2B-16 on day 0 followed by an injection of LPS on day 3. **B**: DBA/1J mice received a new 4-clone cocktail (CII-3, C2B-14, CII-6, and C2A-12, total 2, 4 and 8 mg/mouse) on day 0 followed by LPS. Each value is the mean ± SEM for five animals.

We subsequently added C2A-12 and/or C2B-16 to the 3-clone cocktail (CII-3, CII-6, and C2B-14) to test the arthritogenicity (Figure 2A). The results showed that adding C2A-12 (arthritic score: 10.3 ± 1.0 on day 8) but not C2B-16 (5.0 ± 1.5) to CII-3, CII-6, and C2B-14 was effective in producing more severe arthritis; however, the combination of all 5 mAbs was less effective (arthritic score: 9.2 ± 1.2 on day 8). Furthermore, the severity of the arthritis induced by the combination of CII-3, CII-6, C2B-14, and C2A-12 was dependent on the dose (Figure 2B).

**Figure 3:**
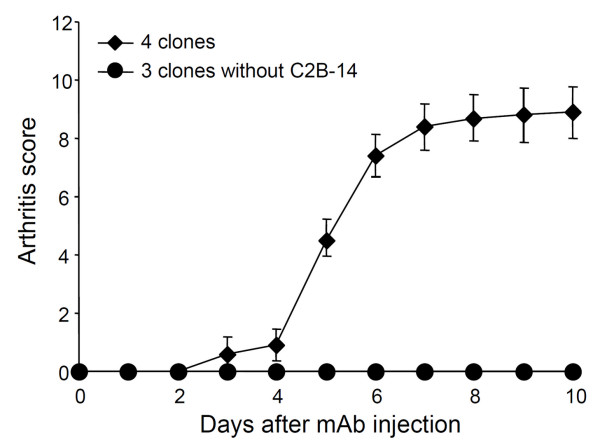
**Effect of excluding C2B-14 on the arthritogenicity of the cocktail (CII-3, C2B-14, CII-6, and C2A-12)**. DBA/1J mice received an injection of 4-clones (CII-3, CII-6, C2B-14, and C2A-12) or 3-clones (CII-3, CII-6, and C2A-12) on day 0 followed by an injection of LPS. Each value is the mean ± SEM for five animals.

Figure 1 shows the importance of C2B-14, without which CII-3, CII-6, and CM-5 showed no arthritogenicity. Thereafter, we examined the effect of excluding C2B-14 from the new cocktail. CII-3, CII-6, and C2A-12 (without C2B-14) caused no arthritis (Figure 3).

### Histological examination of the arthritis induced by the new 4-clone cocktail

**Figure 4:**
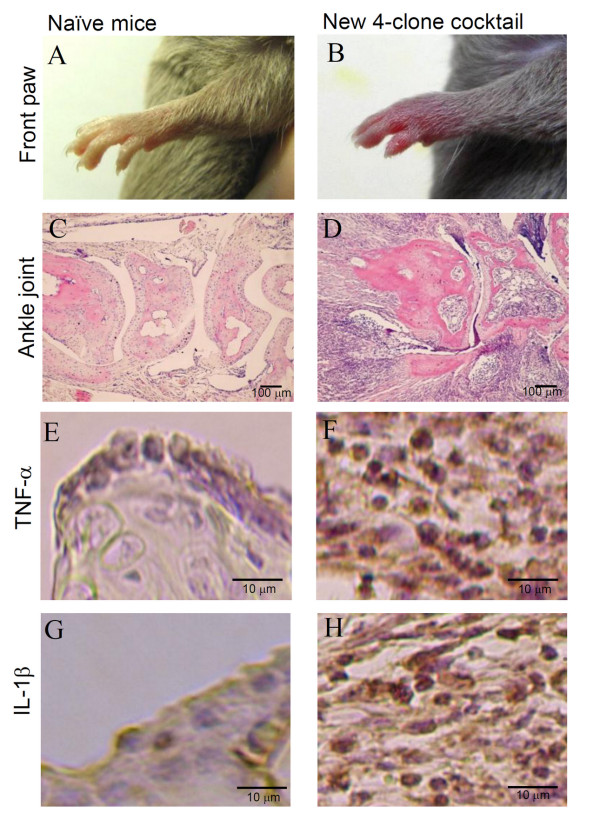
**Histological changes induced by the new 4-clone cocktail (CII-3, CII-6, C2B-14, and C2A-12)**. DBA/1J mice were injected with the new 4-clone cocktail on day 0 followed by LPS. On day 10, the front paws were amputated for histological examination. The tissues were stained with H&E and for immunohistochemistry (TNF-alpha and IL-1beta). Results shown are representative histological pictures of five mice ankle joints in each group. A: normal paw, B: arthritis, C: normal ankle joint, D: arthritic ankle joint, E: normal TNF- alpha, F: arthritic TNF- alpha, G: normal IL-1 beta, H: arthritic IL-1 beta.

Histopathological examination of joints in DBA/1J mice was performed on day 10 after injection of the 4-clone cocktail. Figure 4A and 4C show the naïve front paw and ankle joints as a control, respectively. Mice given the cocktail developed severe arthritis (Figure 4B), and showed marked proliferation of synovial tissues, massive infiltration by inflammatory cells, and severe destruction of cartilage and bone in the ankle joints (Figure 4D).

Figure 4E and 4G show the staining of TNF-alpha and IL-1beta, respectively, in normal joints. Cells expressing TNF-alpha and IL-1beta were detected in inflammatory regions in the treated mice (Figure 4F and 4H).

### Activation of complement and cross-reaction with mouse or chicken CII *in vitro*

**Figure 5:**
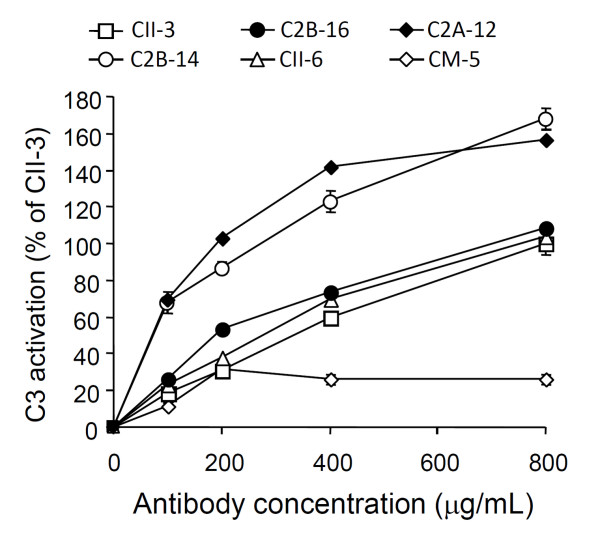
**Activation of complement by mAbs *in vitro***. The activation of C3 by CII-3, C2B-14, C2B-16, CII-6, C2A-12, and CM-5 is shown as a percentage of the CII-3 value (800 μg/ml). Each value is the mean ± SD of four times.

Figure 5 shows the activation of complement by the mAbs as a percentage of the CII-3 value at 800 μg/mL. C2B-14 and C2A-12 showed strong effects compared with the other clones. For example, values for C2B-14 and C2A-12 were 123 and 142% at 400 μg/mL. The levels for C2B-16 (73%) and CII-6 (70%) were similar to that for CII-3 (60%) at 400 μg/mL. On the other hand, complement activation by CM-5 (26%) was less than that by CII-3 at 400 μg/mL. The order of the mAbs in terms of the activation of complement was C2A-12 = C2B-14 > CII-3 = C2B-16 = CII-6 > CM-5.

**Figure 6:**
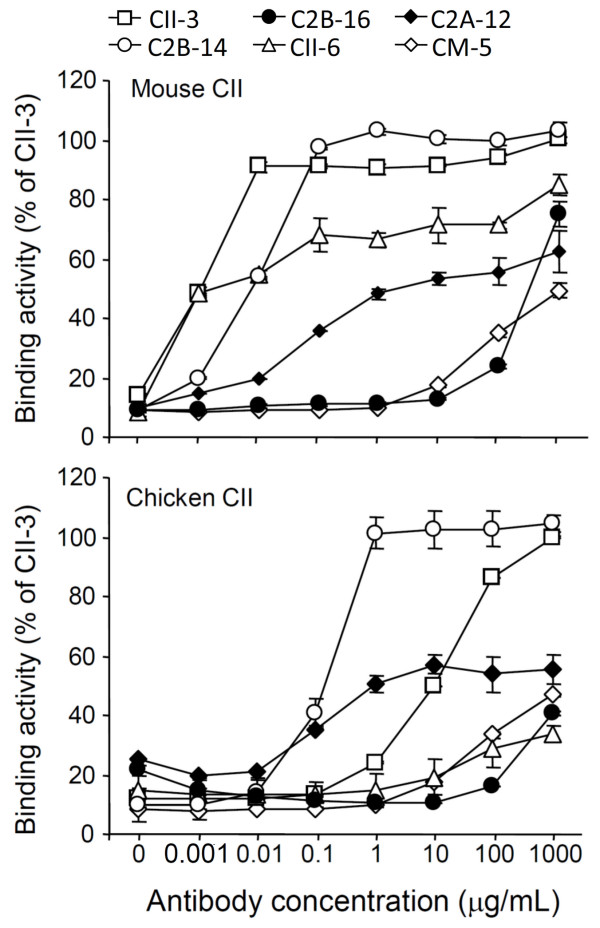
**Cross-reaction of mAbs with mouse or chicken CII**. Increasing concentrations of mAbs (CII-3, C2B-14, C2B-16, CII-6, C2A-12, and CM-5) were analyzed for their ability to bind to mouse or chicken CII-coated plates, and shown as a percentage of the CII-3 value (1000 μg/ml). Each value is the mean ± SD of four times.

Figure 6A and 6B show cross-reaction with mouse and chicken CII, respectively, as a percentage of the CII-3 value at 1000 μg/mL. C2B-14 and CII-3 bound extensively to mouse CII: 103 and 90% at 1 μg/mL, respectively. Furthermore, CII-6 and C2A-12 showed rates of 67 and 48% at 1 μg/mL, respectively; however, C2B-16 and CM-5 did not show binding activity at 1 μg/mL. On the other hand, for chicken CII, CII-6, C2B-16, and CM-5 did not show binding activity at 1 μg/mL, although C2B-14, C2A-12 and CII-3 showed 101, 51 and 24%, respectively. In terms of the cross-reaction of the mAbs with mouse and chicken CII, the order was CII-3 = C2B-14 > CII-6 > C2A-12 > C2B-16 = CM-5, and C2B-14 > C2A-12 > CII-3 > CII-6 = C2B-16 = CM-5, respectively.

## Discussion

The present study demonstrated that a combination of CII-6, CII-3, C2A-12, and C2B-14 induced severe arthritis in DBA/1J mice. Importantly, these 4 anti-CII mAbs showed both marked cross-reactions with mouse CII and the activation of complement, indicating that the initial event in this model is the formation of collagen-antibody immune complexes on the cartilage surface or in the synovium, and subsequent activation of complement by the complexes may induce arthritis.

First, to examine the arthritogenicity of the mAbs, mice were injected with CII-6, CII-3, C2B-14, and CM-5, resulting in mild arthritis. Excluding CM-5 from the cocktail induced a more severe form of arthritis. Furthermore, experiments *in vitro *showed that the activation of complement and binding to mouse CII of CM-5 were much less extensive than with the other clones, suggesting that the development of arthritis was dependent on the characteristics of these mAbs.

Next, we added C2A-12 and/or C2B-16 to the mAbs (CII-6, CII-3, and C2B-14) to test the arthritogenicity. We predicted that C2A-12 would exacerbate the arthritis because its ability to activate complement and bind to mouse CII is greater than that of C2B-16. As expected, adding C2A-12 to the 3-clone cocktail produced more severe arthritis. On the other hand, excluding C2B-14 from the cocktail of CII-6, CII-3, C2B-14 and C2A-12 caused no arthritis because C2B-14 had a greater ability to bind to mouse CII and activate complement among the clones, indicating that C2B-14 is a key factor in this CAIA model, and this *in vitro *system may be useful for the selection of mAbs associated with the development of arthritis.

It has been reported that anti-CII antibodies, including IgG2a and IgG2b, which are complement-fixing isotypes, are a major component in the case of CIA, and their levels are higher at the peak of arthritis [1,21,22]. The newly developed 4-clone cocktail contained 2 IgG2a and 2 IgG2b, suggesting that IgG2a and IgG2b are important antibodies for developing a CAIA model. On the other hand, Nandakumar et al. [23] reported that IgG1 is associated with the development of a CAIA model, suggesting that the addition of an IgG1 mAb to the new cocktail might induce greater arthritis. The relationship between IgG1 mAbs and the development of arthritis needs to be elucidated.

It is thought that complement fragments binding to immune complexes, tissue damage, and/or Fc-gamma receptor crosslinking can activate local mononuclear cells that in turn release proinflammatory cytokines (IL-1beta, TNF-alpha etc) in or near the joints inducing neutrophil and macrophage recruitment [1,24]. Furthermore, in the 4-clone cocktail-treated mice, massive infiltration by inflammatory cells and severe destruction of cartilage and bone in the ankle joints were observed; therefore, we examined whether the new cocktail generated the production of IL-1beta and TNF-alpha in the joints of this CAIA model. IL-1beta and TNF-alpha levels increased on day 10 after the administration of the cocktail, and increases were observed in the inflammatory regions, suggesting that proinflammatory cytokines induce the accumulation of inflammatory cells (macrophages and neutrophils). Furthermore, it was reported that tissue-degrading enzymes of macrophages and neutrophils can cause cartilage and/or bone damage [1], suggesting that the destruction of cartilage and bone in this CAIA model is associated with the accumulation of inflammatory cells.

Autoantibody epitopes located within CB11 play an important role in the development of mouse CIA [16], and two clones (CII-3 and CII-6) of the new cocktail recognize LyC1 of CB1; however, the epitopes of the other two clones (C2A-12 and C2B-14) are unknown, suggesting that the characteristics of the mAbs should be analyzed further.

In conclusion, a combination of four mAbs showing both strong cross-reactions with mouse CII and marked activation of complement effectively induced arthritis in DBA/1J mice. Furthermore, this *in vitro *system may be a useful tool for the selection of mAbs associated with the development of arthritis.

## Competing interests

The authors declare that they have no competing interests.

## Authors' contributions

All authors participated in the design of this study. TK, TK and PH performed hybridoma cell development, hybridoma cell culture and CAIA in the animal model. TK and TK carried out most of the *in vitro *experiments. TK, NM and SY participated in the coordination of the study and manuscript preparation. All authors read and approved the manuscript.
